# Causal Relationship between Mitochondrial Biological Function and Periodontitis: Evidence from a Mendelian Randomization Study

**DOI:** 10.3390/ijms25147955

**Published:** 2024-07-21

**Authors:** Huan Zhou, Yan-Xin Qi, Ruo-Yan Cao, Xi-Xuan Zhang, Ang Li, Dan-Dan Pei

**Affiliations:** 1 Key Laboratory of Shaanxi Province for Craniofacial Precision Medicine Research, College of Stomatology, Xi’an Jiaotong University, Xi’an 710004, China; 2 Department of Periodontology, College of Stomatology, Xi’an Jiaotong University, Xi’an 710004, China; 3 Department of Digital Oral Implantology and Prothodontics, College of Stomatology, Xi’an Jiaotong University, Xi’an 710004, China; 4 Department of Periodontics, Liaoning Provincial Key Laboratory of Oral Diseases, School and Hospital of Stomatology, China Medical University, Shenyang 110002, China

**Keywords:** periodontitis, mitochondria, Mendelian randomization, genome-wide association studies

## Abstract

A growing number of studies indicate that mitochondrial dysfunction serves as a pathological mechanism for periodontitis. Therefore, this two-sample Mendelian randomization (MR) study was carried out to explore the causal associations between mitochondrial biological function and periodontitis, because the specific nature of this causal relationship remains inconclusive in existing MR studies. Inverse variance weighting, Mendelian randomization-Egger, weighted mode, simple mode, and weighted median analyses were performed to assess the causal relationships between the exposure factors and periodontitis. The results of the present study revealed a causal association between periodontitis and medium-chain specific acyl-CoA dehydrogenase (MCAD), malonyl-CoA decarboxylase (MLYCD), glutaredoxin 2 (Grx2), oligoribonuclease (ORN), and pyruvate carboxylase (PC). Notably, MCAD and MLYCD are causally linked to periodontitis, and serve as protective factors. However, Grx2, ORN, and PC function as risk factors for periodontitis. Our study established a causal relationship between mitochondrial biological function and periodontitis, and such insights may provide a promising approach for treating periodontitis via mitochondrial regulation.

## 1. Introduction

Periodontitis is a prevalent infectious disease characterized by biofilm-induced and host mediated inflammation that progressively leads to the destruction of connective tissues, tooth loss and, eventually, jawbone resorption and edentulism. It represents the sixth most prevalent disease worldwide and affects up to 90% of the global population [1,2,3]. Additionally, periodontitis is epidemiologically related to various chronic disorders, including cardiovascular disease, Alzheimer’s disease, type 2 diabetes mellitus, nonalcoholic fatty liver disease, inflammatory bowel disease, rheumatoid arthritis, and certain cancers, which seriously affect the quality of life and account for a vast burden of healthcare cost [4,5]. Thus, the prevention and treatment of periodontitis are difficult problems that society as a whole needs to address [6].

At present, common clinical periodontal treatment strategies, including supragingival scaling, subgingival scaling and root planing, bone transplantation, and guided tissue regeneration, are mainly focused on controlling inflammation and terminating periodontal tissue damage [7]. Although these methods, to a certain degree, can control the clinical progression of periodontitis, reconstruction of the damaged periodontal tissues has remained the ultimate goal of periodontal treatment, and it is also a difficult task in clinic. In this context, further research into the etiology of periodontitis and its progression mechanisms, the identification of novel therapeutic targets, and the design of efficacious treatment measures are of great significance.

Mitochondria are the primary site for the synthesis of adenosine triphosphate (ATP), the main energy source for physiological and biochemical processes. Accumulating research has indicated the vital role of mitochondrial dysfunction during the initiation and progression of periodontitis; more specifically, the excessive production of reactive oxygen species (ROS), alterations in mitochondrial biogenesis and dynamics, mitophagy and mitochondrial DNA damage, can strongly affect the development and progression of periodontitis [8,9]. Mitochondria are the main site for ROS production, and studies have demonstrated that the excessive generation of ROS causes abnormalities in the mitochondrial electron transfer chain and much more ROS release; consequently, excessive ROS can cause oxidative stress, resulting in various disorders, including periodontal tissue damage [10,11,12]. A previous study demonstrated that a hybrid of indole and dithiocarbamate protected against periodontitis by restoring osteoclast mitochondrial function, in terms of mitochondrial ROS, mitochondrial membrane potential, and ATP production [13]. In addition, recent studies have verified that mitochondrial dysfunction might be a vital driver of the reciprocal comorbidity between periodontitis and type 2 diabetes mellitus [14]. Despite continuous research, a comprehensive theoretical framework illustrating the causal link between mitochondrial biological function and periodontitis has yet to be established.

Mendelian randomization (MR) studies are an epidemiological approach that leverages genome-wide association study (GWAS) data and employs genetic variations as instrumental variables (IVs) to assess causal relationships between exposures and outcomes. MR can mitigate the effect of confounding factors and address reverse causation and inference, and its basis on large sample studies facilitates more robust results [15,16]. Hence, the present study was performed, employing MR to assess whether a causal link exists between mitochondrial biological function and periodontitis, with the aim of providing valuable insights into the treatment of periodontitis by regulating mitochondrial function.

## 2. Results

### 2.1. Dataset Screening

Five SNP datasets were strongly associated with periodontitis: ebi-a-GCST90019404 (medium-chain specific acyl-CoA dehydrogenase (MCAD); mitochondrial measurement) [17], prot-a-1907(malonyl-CoA decarboxylase (MLYCD), mitochondrial measurement), prot-a-1220 (glutaredoxin-2 (Grx2); mitochondrial measurement), prot-a-2526 (oligoribonuclease, (ORN); mitochondrial measurement) and prot-a-2190 (pyruvate carboxylase (PC); mitochondrial measurement) [18]. In addition, mitochondrial DNA copy number (mtDNA-CN) was demonstrated to reflect the ratio of mitochondrial to nuclear DNA copy number and can function as a rough substitute to reflect mitochondrial biological function [19,20]. MR analysis of mtDNA-CN and periodontitis was also performed (ebi-a-GCST90026372, mtDNA-CN, mitochondrial measurement), although there was no statistically significant difference [21].

### 2.2. Selection of Instrumental Variables

The “TwoSampleMR” package was used to explore SNPs showing robust relationship between mitochondrial biological function and periodontitis. Specifically, “ebi-a-GCST90019404” exhibited 18 SNPs with robust periodontitis associations, “prot-a-1907” featured 9 SNPs exhibiting robust associations with periodontitis, “prot-a-1220” presented 13 SNPs showing robust associations with periodontitis, 11 SNPs in “prot-a-2526” displayed strongly associations with periodontitis, and 9 SNPs within “prot-a-2190” demonstrated robust associations with periodontitis. For mtDNA-CN, 101 independent periodontitis-related SNPs were screened from the ebi-a-GCST90026372 database (Table 1).

### 2.3. Results of MR Analysis

**Figure 1:**
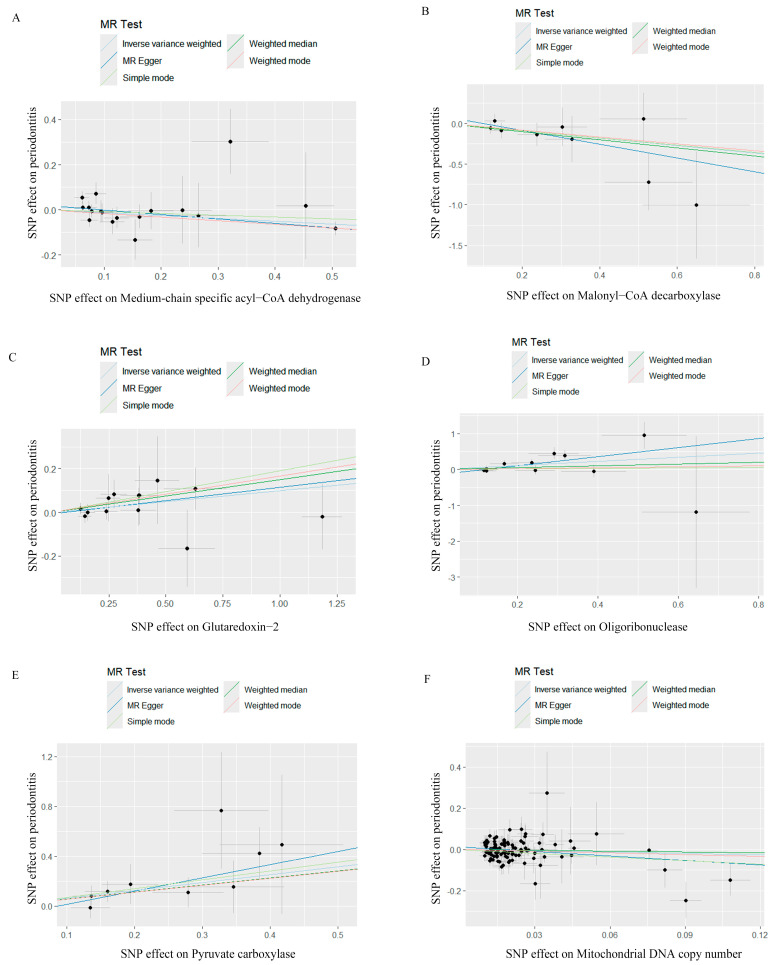
Scatter plots for Mendelian randomization analyses of the causal effect of mitochondrial biological function on periodontitis. (**A**) Scatter plot of the causal relationship between medium−chain specific acyl−CoA dehydrogenase and periodontitis, evaluated by the IVW method. (**B**) Scatter plot of the causal relationship between malonyl−CoA decarboxylase and periodontitis, primarily evaluated via the IVW method. (**C**) Scatter plot of the causal relationship between glutaredoxin−2, mitochondrial and periodontitis, primarily evaluated using the IVW method. (**D**) Scatter plot of the causal relationship between oligoribonuclease and periodontitis using the IVW method. (**E**) Scatter plot of the causal relationship between pyruvate carboxylase and periodontitis, primarily evaluated with the IVW method. (**F**) Scatter plot of the causal relationship between mitochondrial DNA copy number and periodontitis, evaluated using the IVW method. IVW, inverse variance weighting.

**Figure 2:**
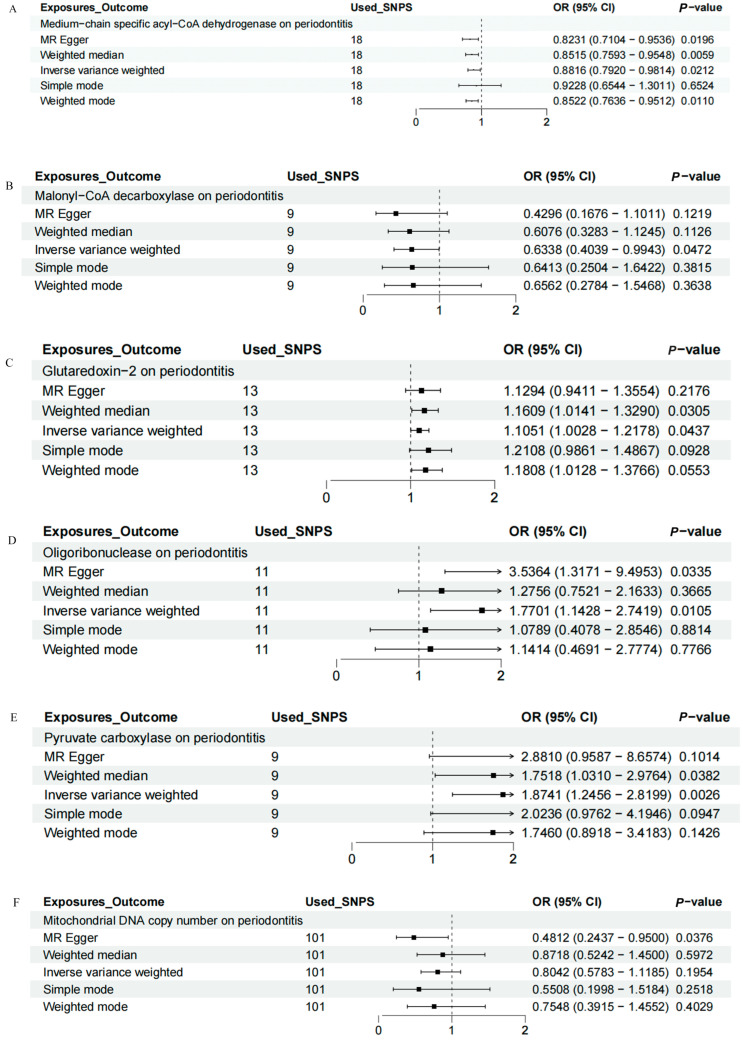
Forest map of the causal relationship between mitochondria and periodontitis (IVW method). (**A**) Forest map of the causal relationship between medium−chain specific acyl−CoA dehydrogenase and periodontitis. (**B**) Forest map of the causal relationship between malonyl−CoA decarboxylase and periodontitis. (**C**) Forest map of the causal relationship between glutaredoxin − 2 and periodontitis. (**D**) Forest map of the causal relationship between oligoribonuclease and periodontitis. (**E**) Forest map of the causal relationship between pyruvate carboxylase and periodontitis. (**F**) Forest map of the causal relationship between mitochondrial DNA copy number and periodontitis. IVW, inverse variance weighting.

IVW, MR-Egger, weighted median, weighted mode, and simple mode analyses were performed to evaluate the causal relationship between mitochondrial function and periodontitis. The MR results were conducted and visualized via the “TwoSampleMR” package in R software (version 4.3.1), as shown in the scatter plots (Figure 1) and forest plots (Figure 2). Based on the IVW results, MCAD (OR = 0.8816, 95% CI = 0.7920–0.9814, *p* = 0.0212) and MLYCD (OR = 0.6338, 95% CI = 0.4039–0.9943, *p* = 0.0472) significantly reduced the risk of periodontitis, acting as protective factors. Grx2 (OR = 1.1051, 95% CI = 1.0028–1.2178, *p* = 0.0437), ORN (OR = 1.7701, 95% CI = 1.1428–2.7419, *p* = 0.0105) and PC (OR = 1.8741, 95% CI = 1.2456–2.8199, *p* = 0.0026) functioned as risk factors for periodontitis, increasing its incidence. There was no causal relationship between mtDNA-CN and periodontitis (OR = 0.8042, 95% CI = 0.5783–1.1185, *p* = 0.1954). 

### 2.4. Sensitivity Analysis

**Figure 3:**
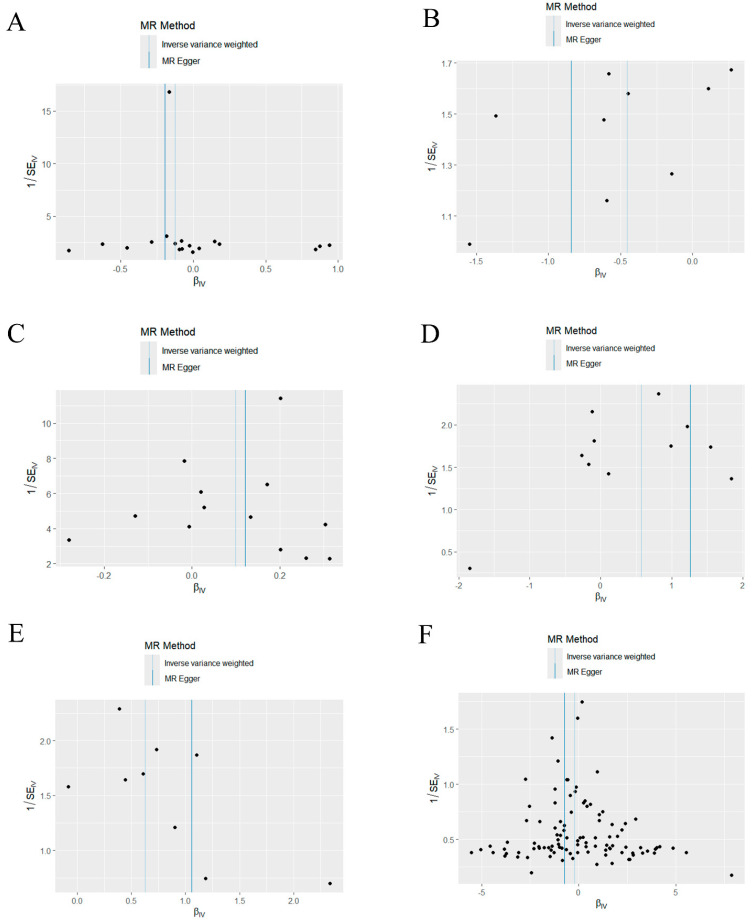
Funnel plot of the causal relationship between mitochondria and periodontitis (IVW method). (**A**) Funnel plot of the causal relationship between medium−chain specific acyl−CoA dehydrogenase and periodontitis. (**B**) Funnel plot of the causal relationship between malonyl-CoA decarboxylase and periodontitis. (**C**) Funnel plot of the causal relationship between glutaredoxin− 2 and periodontitis. (**D**) Funnel plot of the causal relationship between oligoribonuclease and periodontitis. (**E**) Funnel plot of the causal relationship between pyruvate carboxylase and periodontitis. (**F**) Funnel plot of the causal relationship between mitochondrial DNA copy number and periodontitis. IVW, inverse variance weighting.

**Figure 4:**
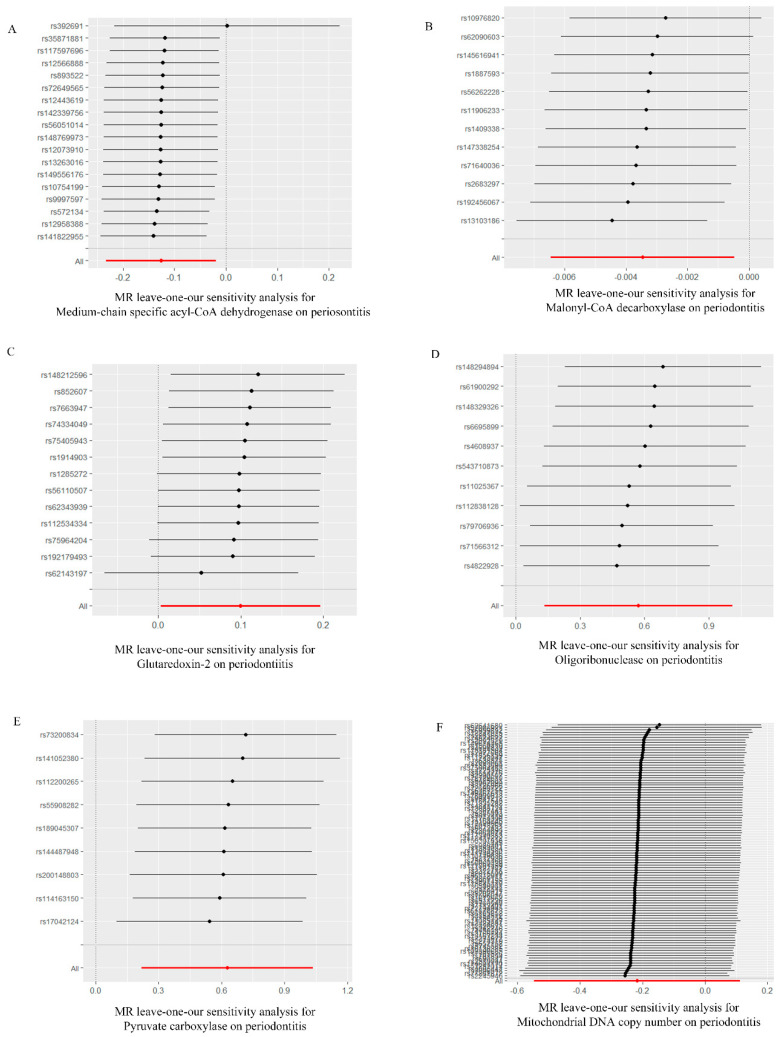
“Leave−one−out” forest map of the causal relationship between mitochondria and periodontitis (IVW method). (**A**) “Leave−one−out” forest map of the causal relationship between medium−chain specific acyl−CoA dehydrogenase and periodontitis. (**B**) “Leave−one−out” forest map of the causal relationship between malonyl−CoA decarboxylase and periodontitis. (**C**) “Leave−one−out” forest map of the causal relationship between glutaredoxin−2 and periodontitis. (**D**) “Leave−one−out” forest map of the causal relationship between oligoribonuclease and periodontitis. (**E**) “Leave−one−out” forest map of the causal relationship between pyruvate carboxylase and periodontitis. (**F**) “Leave−one−out” forest map of the causal relationship between mitochondrial DNA copy number and periodontitis. IVW, inverse variance weighting.

Based on the MR-Egger regression and IVW analysis results, there was no heterogeneity in any of the datasets (*p* > 0.05). Additionally, Egger regression analysis demonstrated that directional horizontal pleiotropy did not occur, as the results revealed *p* > 0.05 across all datasets (Table 2). In addition, the funnel plot showed a symmetrical distribution of SNPs, emphasizing the relative stability of the results (Figure 3). Subsequently, a leave-one-out method was applied to assess the effect of individual SNPs, and the results showed no significant effect on the effect sizes (Figure 4).

## 3. Discussion

Compelling evidence has demonstrated the vital role of mitochondrial dysfunction in periodontitis; therefore, targeted mitochondrial therapy is potentially an effective strategy in periodontitis treatment [22,23,24]. In the present study, we demonstrated that there was a causal relationship between mitochondrial biological function and periodontitis, and discerned that MCAD, MLYCD, Grx2, ORN, and PC are significantly associated with periodontitis. 

MCAD is a mitochondrial enzyme that is involved in the catalysis of fatty acid β-oxidation, a process that is important for the maintenance of energy homeostasis [25,26]. A deficiency in MCAD was demonstrated to affect fatty acid β-oxidation, resulting in lipid deposition in multiple organs. Previous studies have shown that empagliflozin can effectively reduce lipid deposition and ameliorate nonalcoholic steatohepatitis via activating AMPK/FOXA2 signaling to upregulate MCAD expression; thus, MCAD may be a potential therapeutic target for the treatment of nonalcoholic steatohepatitis [27]. Acetyl-CoA by sodium octanoate (8C) was demonstrated to markedly improve heart function in ischemia reperfusion rats and, more importantly, MCAD was verified to be a pivotal factor in the pathophysiology of reperfusion injury mediated by 8C [28]. Based on the obtained results, our findings indicated that MCAD may act as a protective element against periodontitis. MLYCD is a mitochondrial enzyme that catalyzes the breakdown of propionyl-CoA into acetyl-CoA and carbon dioxide, offering a metabolic pathway for propionyl-CoA in mitochondria [29]. Accumulating research has demonstrated that mutations in the *MLYCD* gene can result in a rare inherited metabolic disorder, which manifests as hypoglycemia, metabolic acidosis, and/or cardiomyopathy [30,31]. In addition, MLYCD was found to be downregulated in clear cell renal cell carcinoma (ccRCC), and low MLYCD expression was correlated with a poor prognosis in patients. However, overexpressing MLYCD was verified to effectively reduce tumor growth and reverse resistance to sunitinib; this was because MLYCD-mediated fatty acid oxidation can disrupt endoplasmic reticulum and mitochondrial homeostasis, indicating that MYLCD activation could be a promising strategy for treating ccRCC [32]. Additionally, MYLCD was demonstrated to be involved in the process by which lipid-induced mitochondrial stress contributes to skeletal muscle insulin resistance [33]. Our current study also indicated that MYLCD may be a critical mediator in the development of periodontitis, and may be used as a potential protective agent against periodontitis in the clinic. 

Grx2 is an oxidoreductase located in mitochondria that plays a crucial role in the regulation of mitochondrial redox [34]. Previously, Grx2 was proved to protect lens epithelial cells from oxidative stress-related epithelial–mesenchymal transition via suppressing mitochondrial oxidative stress-related upregulation of integrin-linked kinase [35]. Of note, the lack of Grx2 in mitochondria led to changes in metabolic phenotype, as evidenced by increased body weight, the accumulation of fat in the liver, and augmented plasma lipid levels, which resulted in fatty liver diseases [36]. Based on our results, the expression of Grx2 is positively correlated with periodontitis, which may shed light on the pathological and physiological processes of periodontitis, and provide a potential therapeutic target for the treatment of periodontitis. Based on the current understanding, ORN is an enzyme that is involved in the terminal step of RNA digestion and deletion of ORN can affect pathways regulated by c-di-GMP signaling, for example, the response to oxidative stress [37]. ORN deletion can significantly weaken the motility of *P. aeruginosa*, reduce energy metabolism and adversely affect bacterial chemotaxis [38]. Our research corroborates this view, suggesting that ORN is positively correlated with periodontitis and may promote the occurrence and development of periodontitis by regulating mitochondrial function. PC is a mitochondrial enzyme that contributes to the ATP-dependent conversion of pyruvate to oxaloacetate. Previously, PC was identified to be present in some neurons in the human brain cortex, and further research verified that PC has important functions in the metabolomic cooperation between neurons and astrocytes, which are of importance in understanding the intercellular metabolism of neurons and astrocytes [39]. E.S. Selen et al. proved that PC is vital for mitochondrial pyruvate metabolism, because PC was involved in the high fat, ketogenic diet and fasting processes [40]. An adipocyte-specific lncRNA, ADIPINT, was demonstrated to regulate lipid metabolism by interacting with PC, and further studies revealed that reduced ADIPINT or PC expression can decrease the synthesis and content of lipids in adipocytes [41]. Thus, our results may unveil a novel gene that connects mitochondrial biological function with periodontitis. Additionally, the effect of mtDNA-CN on periodontitis was assessed and, based on the obtained results, there was no causal relationship between mtDNA-CN and periodontitis. Currently, a variety of studies have indicated that mitochondrial dysfunction serves as a pathological mechanism for periodontitis, and therapy based on regulating mitochondria may be an effective method for treating periodontitis [42,43,44]. Our study based on GWAS data indicates a causal association between mitochondrial biological function and periodontitis, and MCAD and MLYCD are causally linked to periodontitis, serving as protective factors, while, Grx2, ORN, and PC function as risk factors for periodontitis. These results may provide therapeutic targets for periodontitis treatment based on mitochondrial therapy.

MR research that leverages GWAS data can, to a certain degree, control confounding factors within observational studies and be used to provide accurate and stable estimates. In addition, potential biases were mitigated effectively by employing LD analysis, association analysis, and weak IVs eliminations [45,46]. Additionally, a symmetrical distribution of SNPs in the funnel plot was observed within our studies. Nevertheless, the present study also has several limitations. First, the present study focused mainly on the European population rather than the Asian and African populations, which may lead to racial biases. Second, the number of IVs for SNPs within this study was relatively modest; thus, it is necessary to utilize larger sample datasets to further validate the results in subsequent analyses. Third, although the causal relationship between mitochondria and periodontitis was explored via MR, it is unclear whether mitochondrial dysfunction influences periodontitis onset or its progress, as the underlying mechanisms were not elucidated, and more in-depth research is needed.

## 4. Materials and Methods 

### 4.1. Study Design

**Figure 5:**
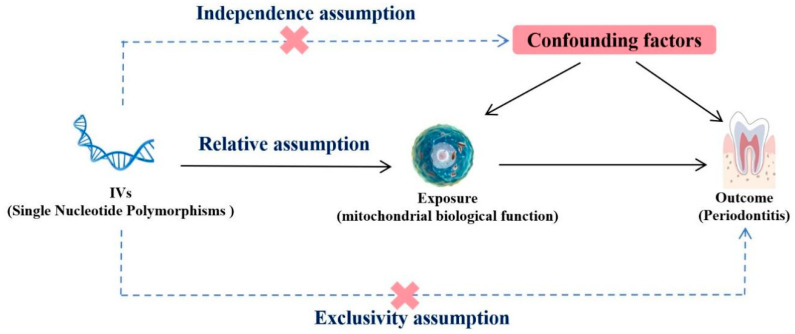
Mendelian randomization design of the present study.

The present MR analysis was predicated on three fundamental assumptions, as shown in Figure 5. The initial assumption was that genetic IVs exhibit a robust association with mitochondria (the exposure). The second assumption was that IVs and periodontitis (the outcome variables) remain unaffiliated with any other confounding factors. The third assumption was that IVs solely impact periodontitis by regulating mitochondrial activity, without any additional pathways.

### 4.2. Genetic Instrument Selection

We employed GWAS data pertaining to mitochondrial biological function as an exposure variable. The selection of IVs associated to mitochondrial function within this study was based on three genome-wide analyses. A total of 10,708 cases and 15,566,792 controls (average age 48.6 years old) were reported by M. Pietzner et al. [17]. A total of 3301 cases and 10,534,735 controls (aged ≥18 years old) were reported by B.B. Sun et al. [18], and 383,476 samples (aged 40–69 years) were reported by M. Chong et al. [21]. All of the above studies collected data based on the European population, and mixed populations or non-European populations were excluded (Table 3). For the present study, summary data can be accessed at https://gwas.mrcieu.ac.uk (accessed on 1 May 2024). Single nucleotide polymorphisms (SNPs) associated with mitochondrial function that met the significance threshold of *p* < 5 × 10^−6^ were selected as potential IVs. In addition, a clumping procedure was performed to set the linkage disequilibrium (LD) coefficient R2 to less than 0.001 in a 10,000 kb window, to determine the independent SNPs. Additionally, F statistical analysis was utilized to assess the strength of SNPs, with F < 10 indicating a possible weak tool. Ambiguous SNPs were deleted to ensure effectiveness and stability.

### 4.3. Outcome Data Source

The GWAS data for periodontitis were selected from the FinnGen database (https://www.finngen.fi/en, accessed on 1 May 2024). The latest whole-genome sequencing results of periodontitis were used in the MR analysis, which included 195,395 controls and 3413 individuals who were diagnosed with periodontitis (acute periodontitis: 367, chronic periodontitis: 3046; the original data are classified according to the 1999 classification of periodontal diseases and conditions).

### 4.4. MR Analysis

The causal relationship between mitochondrial function and periodontitis was determined via the following five methods: inverse variance weighting (IVW), MR-Egger, weighted median, weighted mode, and simple mode. Typically, the IVW method was primarily utilized because of its high statistical efficiency and common application, with a significance threshold of *p* < 0.05 representing a positive result, which is ideal for large samples [47]. The other four robust methods were utilized as complementary methods. MR-Egger can provide a reliable and unbiased estimate, even if all of the SNPs in the selection are invalid [48]. Weighted median can produce reliable estimates of the causal effects, even if as much as 50% of the data come from variations of interest that are invalid IVs [49]. The weighted mode approach maintains validity, even if other instrumental variables do not qualify for the causal inference of the MR technique [50]. Although the simple mode is not as powerful as IVW, this mode-based analysis approach provides robustness to pleiotropy [51]. The results of MR analysis were visualized by the TwoSampleMR package, featuring forest plots, scatter plots, and sensitivity analysis plots.

### 4.5. Sensitivity Analyses

Sensitivity analyses were performed to assess the robustness of our findings. Specifically, MR-Egger and IVW tests were conducted to assess heterogeneity, with a *p* < 0.05 significance level indicating the presence of heterogeneity. In addition, the MR-Egger intercept test was performed to assess the pleiotropy of our findings, and a *p*-value < 0.05 denoted the presence of pleiotropy. For the sensitivity analysis, the “leave-one-out” method was employed to observe the influence of individual SNPs on the overall estimates.

### 4.6. Statistical Analysis

R software (version 4.3.1, the University of Auckland, Auckland, New Zealand), and the TwoSampleMR software package (version 0.5.11) were used to evaluate causality in MR analysis. A threshold of statistical significance of *p* < 0.05 was adopted.

## 5. Conclusions

The present MR study based on GWAS data indicates a causal association between mitochondrial biological function and periodontitis. Notably, MCAD and MLYCD are causally linked to periodontitis, serving as protective factors. Grx2, ORN, and PC function as risk factors for periodontitis. Future studies into the underlying biological mechanisms between periodontitis and these factors may facilitate the development of strategies aimed at treating periodontitis through mitochondrial regulation.

## Figures and Tables

**Table 1:** Mitochondrial dataset tool variation scale.

GWAS-ID	Type	SNPs
ebi-a-GCST90019404	periodontitis	18
prot-a-1907	periodontitis	9
prot-a-1220	periodontitis	13
prot-a-2526	periodontitis	11
prot-a-2190	periodontitis	9
ebi-a-GCST90026372	periodontitis	101

**Table 2:** Heterogeneity analysis.

GWAS-ID	Type	Heterogeneity Analysis		Directional Horizontal Pleiotropy Test
		MR-Egger	IVW	
ebi-a-GCST90019404	periodontitis	0.3937476	0.3476330	0.2101159
prot-a-1907	periodontitis	0.08085533	0.11513503	0.7229887
prot-a-1220	periodontitis	0.8031630	0.8559007	0.8559007
prot-a-2526	periodontitis	0.9700019	0.8663085	0.1479013
prot-a-2190	periodontitis	0.7997403	0.7766346	0.3516742
ebi-a-GCST90026372	periodontitis	0.2619731	0.2196159	0.09462437

**Table 3:** Mitochondria-related GWAS data.

Name	GWAS-ID	Sample Size	Number of SNPs	Population	Age	Sex	Country	Study Design	PMDIN
Medium-chain specific acyl-CoA dehydrogenase, mitochondrial measurement	ebi-a-GCST90019404	10,708	15,566,792	European	Average 48.6 years old	Males and females	England	Cohort study	33328453
Malonyl-CoA decarboxylase, mitochondrial	prot-a-1907	3301	10,534,735	European					29875488
Glutaredoxin-2, mitochondrial	prot-a-1220	3301	10,534,735	European	≥18 years old	Males and females	England	Interval study	
Oligoribonuclease, mitochondrial	prot-a-2526	3301	10,534,735	European					
Pyruvate carboxylase, mitochondrial	prot-a-2190	3301	10,534,735	European					
Mitochondrial DNA copy number	ebi-a-GCST90026372	383,476	11,173,383	European	40–69 years old	Males and females	England	Cohort study	35023831

## Data Availability

The datasets analyzed during the current study are available in the GWAS Catalog (https://gwas.mrcieu.ac.uk/ (accessed on 13 June 2024)).
